# Effects of the Complex of *Panicum miliaceum* Extract and *Triticum aestivum* Extract on Hair Condition

**DOI:** 10.3390/nu15204411

**Published:** 2023-10-18

**Authors:** Nahyun Choi, Ki Cheon Kim, Pan-Young Jeong, Bumsik Kim

**Affiliations:** 1 Epibiotech Co., Ltd., Incheon 21984, Republic of Korea; nh147837@epibiotech.com; 2 Life Science Research Institute, Novarex Co., Ltd., Cheongju 28220, Republic of Korea; k.kicheon@novarex.co.kr; 3 Department of Food and Nutrition, Yeonsung University, Anyang 14011, Republic of Korea

**Keywords:** complex of millet extract and wheat extract, hair health, anagen elongation, antioxidant, growth factor, functional food ingredient

## Abstract

Proso millet (*Panicum miliaceum* L.) and common wheat (*Triticum aestivum* L.) have been used as major crops in multiple regions since ancient times, and they contain various nutrients that can affect human hair health. This study investigated the various biological effects of a complex of millet extract and wheat extract (MWC) on hair health. Human immortalized dermal papilla cells (iDPCs) for an in vitro study and an anagen-synchronized mouse model for an in vivo study were employed. These findings revealed that the application of the MWC in vitro led to an increase in the mRNA levels of antioxidant enzymes (catalase and SOD1), growth factors (IGF-1, VEGF, and FGF7), and factors related to hair growth (wnt10b, β-catenin) while decreasing inflammatory cytokine mRNA levels (IL-6 and TNFα). The mRNA levels of hair follicles (HFs) in the dorsal skin of the mouse model in the early and late telogen phases were also measured. The mRNA levels in the in vivo study showed a similar alteration tendency as in the in vitro study in the early and late telogen phases. In this model, MWC treatment elongated the anagen phase of the hair cycle. These findings indicate that the MWC can suppress oxidative stress and inflammation and may elongate the anagen phase by enhancing the growth factors involved in the wnt10b/β-catenin signaling pathway. This study suggests that the MWC might have significant potential as a functional food for maintaining hair health.

## 1. Introduction

The study of hair encompasses two main fields of interest: the hair outside of the skin and the hair inside of the skin. The external hair is influenced by post-emergent factors such as UVB radiation, chemical treatments (e.g., hair perming or dyeing), and heat styling (e.g., with hair dryers). However, the internal hair, including hair dermal follicle cells, is affected by pre-emergent factors, such as hormonal imbalances, genetic disorders, nutrient deficiencies, and cancer treatments (e.g., anticancer drugs or irradiation therapy) [1]. The study of internal hair health has primarily focused on the biological aspects, particularly examining factors related to hair loss or growth, ultimately addressing hair conditions [2]. The dermal papilla cells (DPCs) of animals with fur are responsible for hair production in the hair papilla located at the root of the hair follicles (HFs) between the dermal and epidermal layers. Oxidative stress and inflammation can lead to the senescence or apoptosis of DPCs [3,4,5]. DPCs that have undergone senescence or apoptosis can lead to unhealthy hair, but they can be potentially restored using materials with antioxidative and anti-inflammatory properties [6,7,8]. Moreover, growth factors and factors related to hair growth can regulate the formation and regeneration of DPCs and affect parts of the hair cycle (e.g., anagen or telogen phases) [9]. The wnt/β-catenin signaling pathway is well known to promote the hair anagen phase and suppress the telogen phase through various signals [10]. However, the reagents or medicines that directly address hair loss or growth by suppressing dihydrotestosterone (DHT) have adverse effects, such as sexual dysfunction and hormone abnormalities [10,11]. There have been few studies on the functional reagents that can indirectly address hair loss or growth without adverse effects through regulating oxidative stress and inflammation [3,4,5,6,7,8].

Proso millet (*Panicum miliaceum* L.) and common wheat (*Triticum aestivum* L.) have been used as food sources since the Neolithic age, when humans started cultivating crops. Now, they are well-known important crops throughout the world for both animals and humans. Recently, research on the efficacy of millet and wheat grain extracts for human or animal health has been performed, focusing on the effects of antioxidation [12] and anti-inflammation [13,14]. Miliacin, the major material of triterpenoid from millet grains extracted by the supercritical CO_2_ extraction method, can affect hair growth by inducing the proliferation of hair follicle cells (HFCs) [15,16]. Moreover, miliacin can prevent the stress-induced activation of lipid peroxidation [17]. The properties of miliacin are associated with the mechanisms of action to regulate the balance of redox reactions [18]. Miliacin treatment is an efficacious topical treatment for trophic ulcers since miliacin possesses anti-inflammatory properties and stimulates regeneration processes [19]. In addition, miliacin protects against the toxic effects of anti-cancer drugs and antibiotics on organs, such as the spleen and thymus [20,21]. Wheat extract containing polyphenols alleviates inflammation and inhibits oxidative stress by regulating NF-κB and Nrf2 signals [22,23]. Wheat extract containing glucosylceramide has been shown to decrease UVB-induced photoaging in vivo, and the oral administration of wheat glucosylceramide improved skin hydration and age-related symptoms, such as loss of elasticity, roughness, and wrinkles, in a human clinical study [24]. Glucosylceramide, or any cerebroside in which the monosaccharide head group is glucose, is found in the skin tissues of animals, plants, and fungi [25]. The administration of glucosylceramide has ameliorated inflammation in aged mice by inhibiting inflammation [26].

Based on these properties of proso millet and common wheat, a millet extract and wheat extract complex (MWC) was manufactured to identify its hair health effects. This study examined the mRNA levels of antioxidant enzymes and inflammatory cytokines, as well as the growth factors and factors related to hair growth. In an anagen-synchronized mouse model, whether the administration of the MWC elongated the hair anagen phase was also investigated.

## 2. Materials and Methods

### 2.1. MWC Preparation

The MWC was provided by Novarex (Republic of Korea). Proso millet (*P. miliaceum* L.) seeds containing miliacin were extracted using the supercritical CO_2_ extraction process. Sunflower oil (SFO) was added as an excipient to stabilize the content. Common wheat (*T. aestivum* L.) extract containing glucosylceramide was harvested via the ethanol extraction method. We designate miliacin and glucosylceramide as active and indicative compounds. We analyzed the quantities of miliacin and glucosylceramide in the MWC to ensure the quality of the extraction process using GC-FID (gas chromatograph fitted with a flame ionization detector) method and LC-MS (liquid chromatography–mass spectrometry) method, respectively. We employed a 5% phenyl-methylpolysiloxane column (0.25 μm, 0.25 mm × 30 m) in the GC-FID method. The results indicated that the miliacin content in the MWC was quantified at 12 mg/g. In the LC-MS method, we utilized an octadecyl silica column (1.7 μm, 2.1 mm × 100 mm). The analysis revealed that the glucosylceramide content in MWC was measured to be 2.5 mg/g. The MWC was mainly composed of millet extract and wheat extract in a ratio of 5.5:1 (*w*/*w*), respectively.

### 2.2. Cell Culture

Human immortalized dermal papilla cells (human iDPCs, KNU201, Epibiotech Co., Ltd., Incheon, Republic of Korea), immortalized by inserting hTERT and SV40-Tag into DPCs, were used. DPCs were cultured in Dulbecco’s Modified Eagle Medium (DMEM) supplemented with 10% fetal bovine serum (FBS) at 37 °C and 5% CO_2_ in a humidified incubator. Subculture was performed once every 2–3 days, and 0.25% trypsin/10 mM EDTA (Thermo Fisher Scientific, Waltham, MA, USA) was used to detach adherent cells. When subcultured seven times or more, the doubling rate and the DPC biomarker expression rapidly decreased, so all experiments were completed using cells before the seventh subculture.

### 2.3. Chemicals

Dimethyl sulfoxide (DMSO), 100× penicillin-streptomycin, chloroform solution, and eosin Y solution were purchased from Sigma-Aldrich (Saint Louis, MO, USA). A hematoxylin solution and a dihydrotestosterone enzyme-linked immunosorbent assay (ELISA) kit were purchased from Abcam, Inc. (Cambridge, MA, USA). Dulbecco’s phosphate-buffer saline (DPBS) was purchased from Corning, Inc. (Corning, NY, USA). DMEM, 0.25% Trypsin-EDTA, and TRIzol were purchased from Thermo Fisher Scientific, Inc. (Waltham, MA, USA). Follicle DPC growth medium was purchased from PromoCell GmbH (Heidelberg, Germany). EZ-Cytox cell viability assay reagent was purchased from DoGenBio, Inc. (Seoul, Republic of Korea). HelixCript Thermo Reverse Transcriptase and RNase inhibitor were purchased from Nanohelix, Inc. (Daejeon, Republic of Korea). A TB Green^®^ Premix Ex Taq kit was purchased from Takara Korea Biomedical, Inc. (Seoul, Republic of Korea). Collagen type I-coated wells were purchased from SPL, Inc. (Gyenggi, Republic of Korea). Pansidil was purchased from Dongkook Pharmaceutical, Inc. (Seoul, Republic of Korea).

### 2.4. Cell Viability

Human iDPCs were seeded in 96-well plates at a density of 1 × 10^3^ cells/well and incubated overnight in DMEM supplemented with 10% FBS. The next day, 60, 120, and 240 μg/mL of MWC were applied to each well. In addition, they were treated with SFO, used as an excipient, at a concentration of 240 μg/mL. For the cell viability analysis, a water soluble tetrazolium salt (WST) assay kit (EZ-Cytox, DoGenBio, Seoul, Republic of Korea) was used according to the manufacturer’s instructions.

### 2.5. Mouse Breeding and Anagen-Synchronized Mouse Model

Mice were maintained and anesthetized according to the protocols of the US Pharmacopoeia and Yonsei University Institutional Animal Care and Use Committee (approval number: IACUC-202203-1425-01). Forty-eight 6-week-old C3H/HeN male mice were purchased (OrientBio Inc., Gyeonggi, Republic of Korea), separated into eight mice per group based on body weight, and allowed to adapt for three days. Then, 30, 60, and 120 mg/kg of MWC, 120 mg/kg of SFO, and 400 mg/kg of Pansidil were orally administered daily to 6.5-week-old mice during the test period. One week after the initial administration, the dorsal hair was shaved using animal hair clippers, leaving 3 mm of hair to avoid damaging the skin. Subsequently, the hair was completely removed using WAX-ready strips to forcefully synchronize anagen HFs [2,27]. After depilation, hair removal was performed with animal hair clippers in the early telogen phase on Day 23 and the late telogen phase on Day 29 (Figure S1). The amount of residual hair that was not shed was measured. Half of the dorsal skin tissue was used to measure the mRNA levels, and the other half was used to observe anagen HFs through H&E staining.

### 2.6. RNA Extraction and Quantitative RT-PCR

Total RNA was extracted from the dorsal skin tissues of mice using Trizol (Invitrogen, Carlsbad, CA, USA) according to the manufacturer’s extraction method. The extracted RNA was synthesized into cDNA using oligo(dT) and the HelixCript Thermo Reverse Transcription System (Nanohelix, Madison, WI, USA). Gene expression was measured by performing quantitative RT-PCR using cDNA with a TB Green Premix Ex Taq (Takara, San Jose, CA, USA) kit. Target genes that can be used in both humans and mice and the corresponding primer sequences are shown in Table 1.

### 2.7. H&E Staining

For the H&E staining of the skin, paraffin sections were prepared by securing the skin from the epidermis to the dermis layer. The sections were then mounted on individual slides. The paraffin sections on each slide were dewaxed three times for 15 min each using xylene; hydrated in 100%, 90%, 80%, and 70% EtOH for 2 min each; and treated with a hematoxylin solution (Abcam, Cambridge, MA, USA) for about 10 min. The sections were dyed and then washed three to five times with distilled water. The sections were soaked in an eosin Y solution for 90 s and then washed with distilled water three to five times. Stained sections were immersed in 70, 80, 90, and 100% EtOH for 2 min to dehydrate the tissue; immersed three times in new xylene for 10 min each; dried at room temperature; and mounted with a mount solution. After completely solidifying the mount solution, growth and telogen HFs were observed using a phase-contrast optical microscope.

### 2.8. Statistics

All results are expressed as the mean ± standard deviation (SD) and were analyzed using a *t*-test followed by a paired *t*-test and a one-way analysis of variance (ANOVA) followed by Dunnett’s test. Statistical significance was set at a *p*-value less than 0.05. The statistical analysis was carried out using GraphPad Prism v5.01 (GraphPad Software Inc., Boston, MA, USA).

## 3. Results

### 3.1. Effects of MWC on Cell Proliferation in Human iDPCs

**Figure 1:**
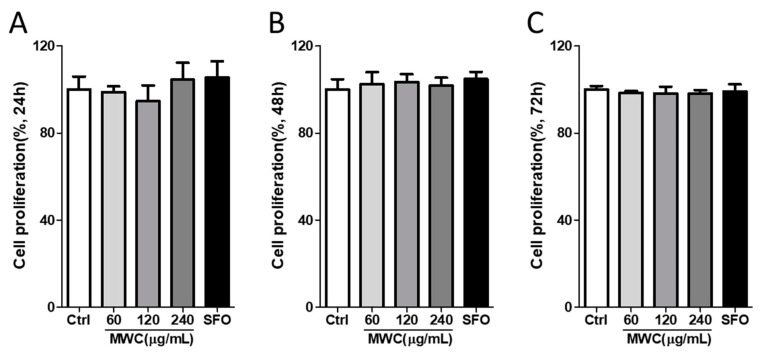
Proliferative effect of MWC on human iDPCs. Cells were treated with 60, 120, or 240 μg/mL of MWC and 240 μg/mL of SFO for (**A**) 24 h, (**B**) 48 h, and (**C**) 72 h. Cell viability was analyzed using a WST assay kit. Each experiment was performed in triplicate. Results were analyzed to assess cell proliferation in the groups treated with MWC or SFO in comparison to control group (ctrl) using a one-way ANOVA.

Cultured human iDPCs were treated with the MWC at different concentrations and observed for 1–3 days. To evaluate the proliferative effect of the MWC on DPCs, the dehydrogenase activities of the cells were determined via a WST analysis after the completion of the culture time. The group treated with the MWC for 24, 48, and 72 h showed no significant difference from the control group, regardless of the concentration (Figure 1). In the group treated with SFO, there was no significant difference compared to the control group at any time. These results confirmed that the MWC had no direct effect on the viability of human iDPCs.

### 3.2. Effect of MWC on Gene Expressions of Antioxidant Enzymes and Inflammatory Cytokines in Human iDPCs

**Figure 2:**
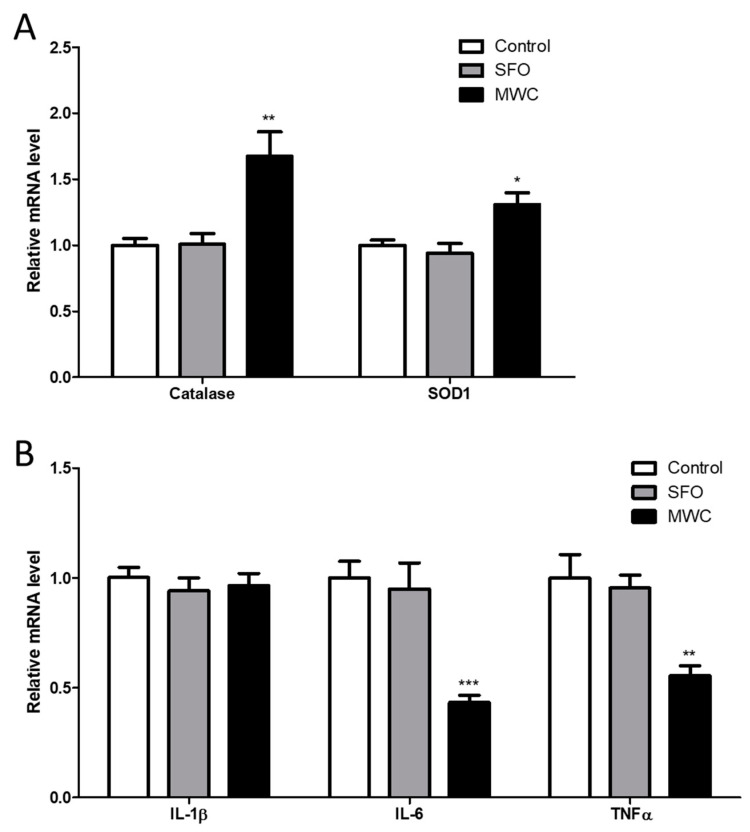
Effect of MWC on the mRNA of antioxidant enzymes and inflammatory cytokines in human iDPCs. Cells were treated with 240 ug/mL of MWC and SFO for 24 h, and the total RNA was extracted. Quantitative RT-PCR was performed to measure the mRNA levels of (**A**) catalase and SOD1 and (**B**) IL-1β, IL-6, and TNFα. Each experiment was performed in triplicate. Results were analyzed using a student’s *t*-test (* *p* < 0.05, ** *p* < 0.01, *** *p* < 0.001 vs. control).

The human iDPCs were treated with high concentrations of the MWC (240 μg/mL) and SFO (240 μg/mL) to determine the effect on the gene expression of antioxidant enzymes (Figure 2A) and inflammatory cytokines (Figure 2B). The mRNA expression of catalase and superoxide dismutase 1 (SOD1), antioxidant enzymes, was significantly increased in the MWC-treated group compared to the control group. In the case of interluekin-1β (IL-1β) mRNA, an inflammatory cytokine, there was no significant difference in the MWC-treated group compared to the control group. The expression of interluekin-6 (IL-6) mRNA and tumor necrosis factor α (TNFα) mRNA was significantly decreased in the MWC-treated group compared to the control group.

### 3.3. Effects of MWC on Growth and Hair-Growth-Related Gene Expression in Human iDPCs

**Figure 3:**
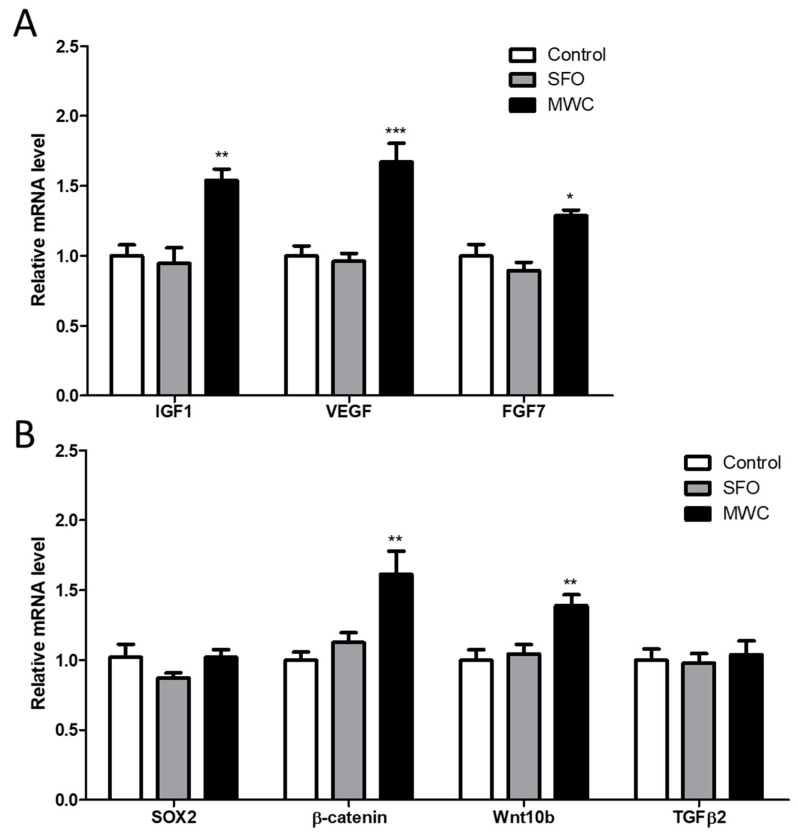
Modulating effect of MWC on the mRNA levels of growth and hair-growth-related factors in iDPCs. Quantitative RT-PCR was performed to measure the mRNA levels of (**A**) IGF1, VEGF, and FGF7 and (**B**) SOX2, β-catenin, wnt10b, and TGFβ2. Each experiment was performed in triplicate. Results were analyzed using a student’s *t*-test (* *p* < 0.05, ** *p* < 0.01, *** *p* < 0.001 vs. control).

To confirm the effect of the MWC on the expression of growth factors and factors related to hair growth in human iDPCs, growth factors such as insulin growth factor-1 (IGF-1), vascular endothelial growth factor (VEGF), and fibroblast growth factor 7 (FGF7), and factors related to hair growth such as the sex-determining regions Y-box 2 (SOX2), β-catenin, wnt10b, and transforming growth factor β2 (TGFβ2) were examined 24 h after the cells were treated with 240 μg/mL of the MWC. The mRNA expression of those factors in the MWC-treated group was compared to those in the control group. The expression of the growth factors (IGF-1, VEGF, and FGF7) was significantly increased in the MWC-treated group compared to the control group (Figure 3A). The mRNA level of SOX2, a hair-growth-related factor, did not show a significant difference in the MWC-treated group compared to the control group (Figure 3B). The mRNA expression of β-catenin and Wnt10b was significantly increased in the MWC-treated group compared to the control group (Figure 3B). TGFβ2 mRNA expression did not show a significant difference between the MWC-treated group and the control group (Figure 3B). There was no significant difference between the SFO-treated group and the control group in the mRNA expression of any of the growth or hair-growth-related factors.

### 3.4. Effects of MWC on Gene Expression of Antioxidant Enzymes and Inflammatory Cytokines in an Anagen-Synchronized Mouse Model

**Figure 4:**
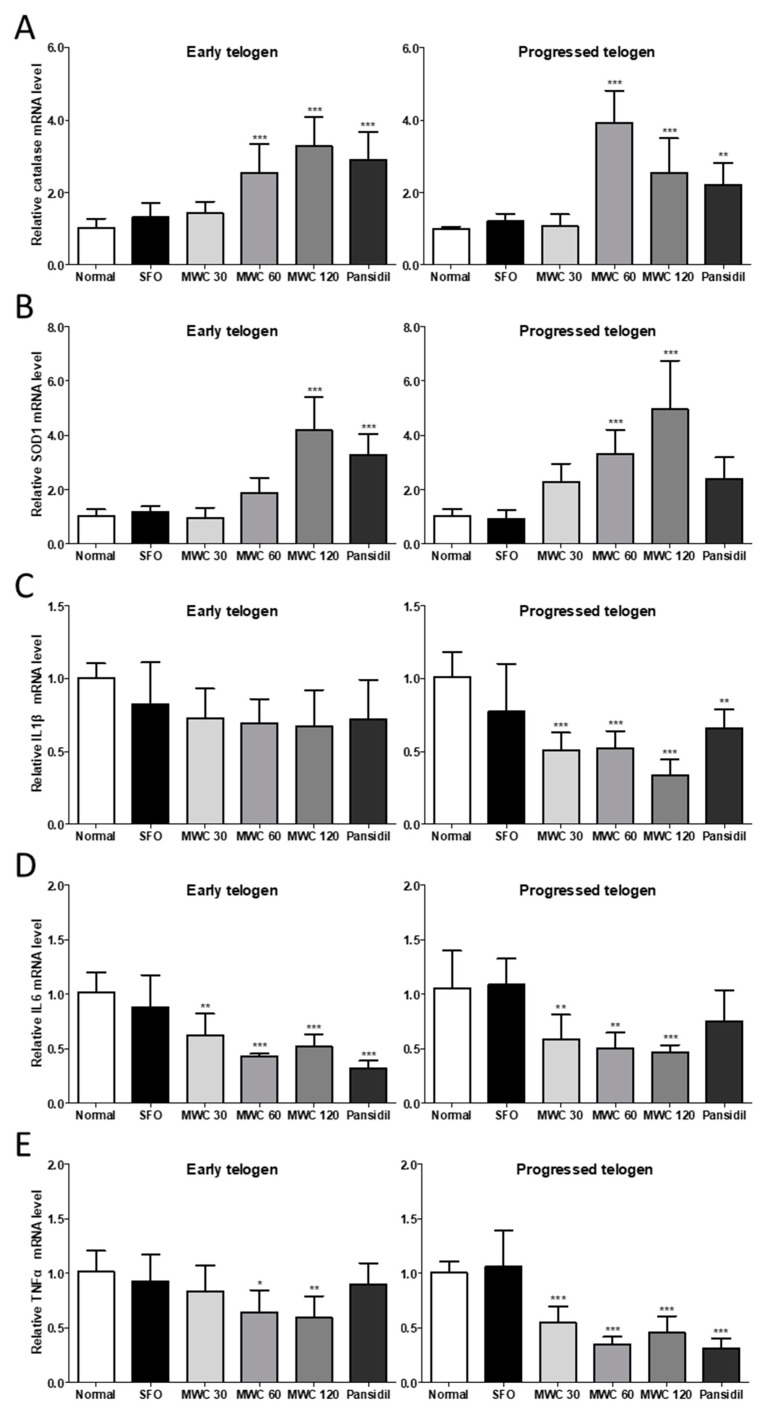
Modulating effect of MWC on the mRNA levels of antioxidant enzymes and inflammatory cytokines in dorsal skin tissues of the anagen-synchronized mouse model. The anagen-synchronized mice were orally administered 30, 60, and 120 mg/kg of MWC (MWC 30, MWC 60, and MWC 120, respectively), 120 mg/kg of SFO, and 400 mg/kg of pansidil following the experimental design. Dorsal skin tissues were harvested in the early and late telogen phases. Total RNA was collected from the dorsal skin tissues, and quantitative RT-PCR was performed to measure the mRNA levels of (**A**) catalase, (**B**) SOD1, (**C**) IL-1β, (**D**) IL-6, and (**E**) TNFα. Each experiment was performed in triplicate. Results were analyzed using a one-way ANOVA (* *p* < 0.05, ** *p* < 0.01, *** *p* < 0.001 vs. control).

The dorsal skin tissues of the early and late telogen phases in the anagen-synchronized mouse model were used to investigate the mRNA levels of antioxidant enzymes and inflammatory cytokines. According to the manufacturer’s instructions, pansidil was utilized as a positive control to promote hair growth and prevent hair loss. The oral administration of the MWC significantly increased the mRNA levels of catalase and SOD1 in a dose-dependent manner in both the early and late telogen HFs of the anagen-synchronized mouse model (Figure 4A,B). The MWC significantly decreased the IL-1β mRNA level in the late telogen HFs in a dose-dependent manner (Figure 4C). The administration of the MWC resulted in dose-dependent decreases in IL6 and TNFα mRNA levels in both the early and late telogen HFs (Figure 4D,E). These results suggest that the oral administration of the MWC might increase the expression of antioxidant enzymes and decrease the expression of inflammatory cytokines.

### 3.5. Effects of MWC on Gene Expression of Growth and Hair-Growth-Related Factors in Anagen-Synchronized Mouse Model

**Figure 5:**
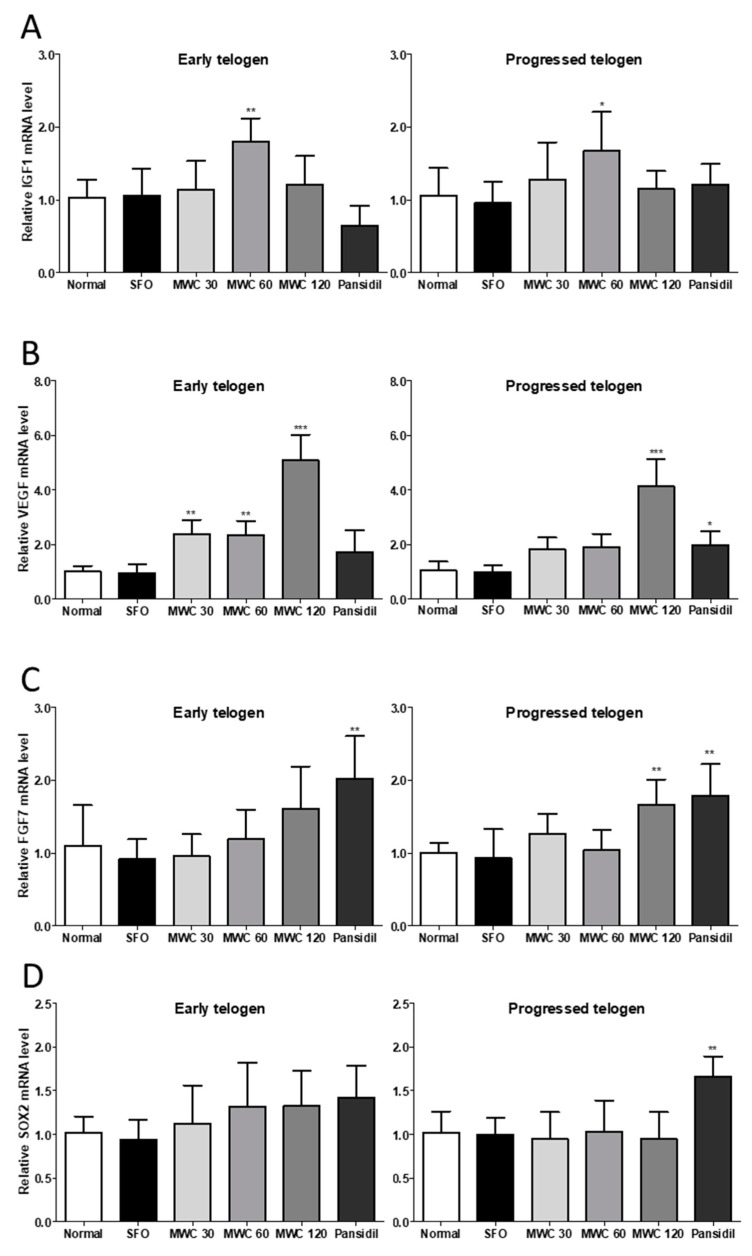

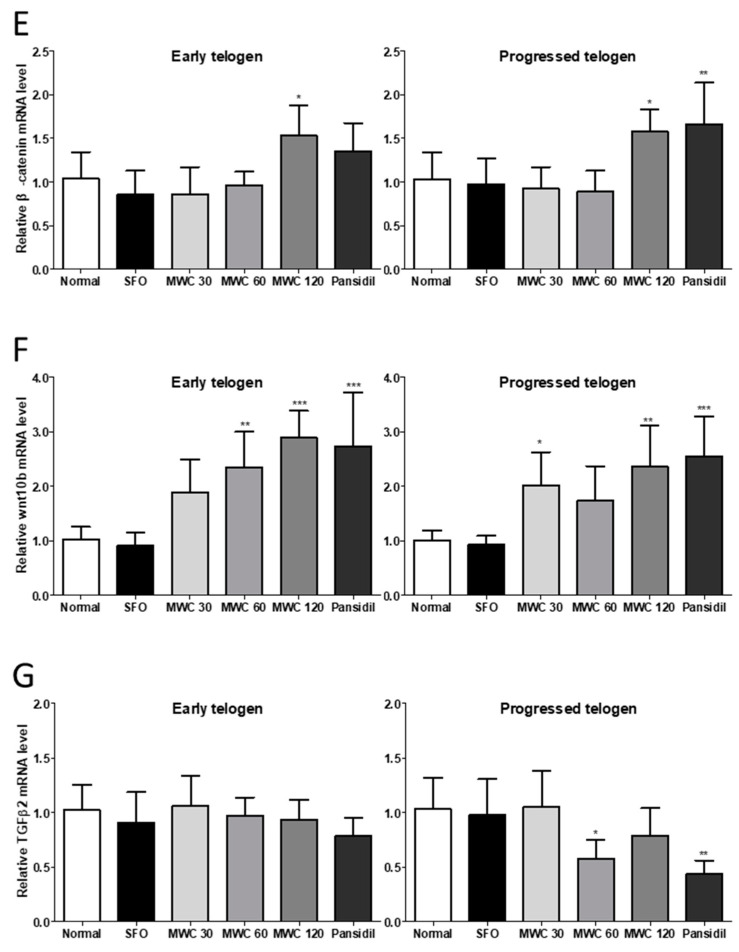
Modulating effect of MWC on the mRNA levels of growth factors and hair-growth-related genes in dorsal skin tissues of the anagen-synchronized mouse model. Dorsal skin tissues were harvested on Day 23 and Day 29. Total RNA was collected from skin tissues, and quantitative RT-PCR was performed to measure the mRNA levels of (**A**) IGF1, (**B**) VEGF, (**C**) FGF7, (**D**) SOX2, (**E**) β-catenin, (**F**) wnt10b, and (**G**) TGFβ2. Each experiment was performed in triplicate. Results were analyzed using a one-way ANOVA (* *p* < 0.05, ** *p* < 0.01, *** *p* < 0.001 vs. control).

The mRNA levels of the growth factors (IGF-1, VEGF, and FGF7) and factors related to hair growth (SOX2, β-catenin, wnt10b, and TGFβ2) were identified in the dorsal skin tissues during the early and late telogen phases. IGF-1 mRNA was significantly increased during the early and late telogen phases in the 60 mg/kg-MWC-administered group compared to the control group (Figure 5A). VEGF mRNA had a concentration-dependent increase after the MWC administration in both the early and late telogen HFs (Figure 5B). The number of HFs in the early telogen phase was significantly increased compared to the control group at all the MWC concentrations, whereas the number of HFs in the late telogen phase was significantly increased only at a concentration of 120 mg/kg compared to the control group. The FGF7 mRNA level was increased in a concentration-dependent manner after the MWC administration in the early telogen HFs but had no significant difference compared to the control group. In the late telogen HFs, the 120 mg/kg-MWC group had a significant increase in the FGF7 mRNA level compared to the control group (Figure 5C). The mRNA level of SOX2, a hair-growth-related gene, showed no significant difference between the MWC-administered group and the control group in either the early or late telogen HFs (Figure 5D). The β-catenin mRNA level was significantly increased in the early and late telogen HFs in the group administered 120 mg/kg of MWC compared to the control group (Figure 5E). The Wnt10b mRNA level had a concentration-dependent increase in the early telogen HFs in the MWC-administered group and a significant increase compared to the control group in the 60 and 120 mg/kg groups. In the late telogen HFs, a concentration-dependent increase was confirmed in the group administered the MWC at concentrations of 30 and 120 mg/kg compared to the control group (Figure 5F). The TGFβ2 mRNA level was significantly decreased in the late telogen HFs in the group administered 60 mg/kg of the MWC compared to the control group; however, there was no significant difference in any of the MWC-administered groups (Figure 5G). In the case of the SFO-administered group, the expression levels of the growth factor and hair-growth-related genes were not significantly different from those of the control group, regardless of the telogen phase.

### 3.6. Effects of MWC on the Elongation of Hair during the Anagen Phase in the Anagen-Synchronized Mouse Model

**Figure 6:**
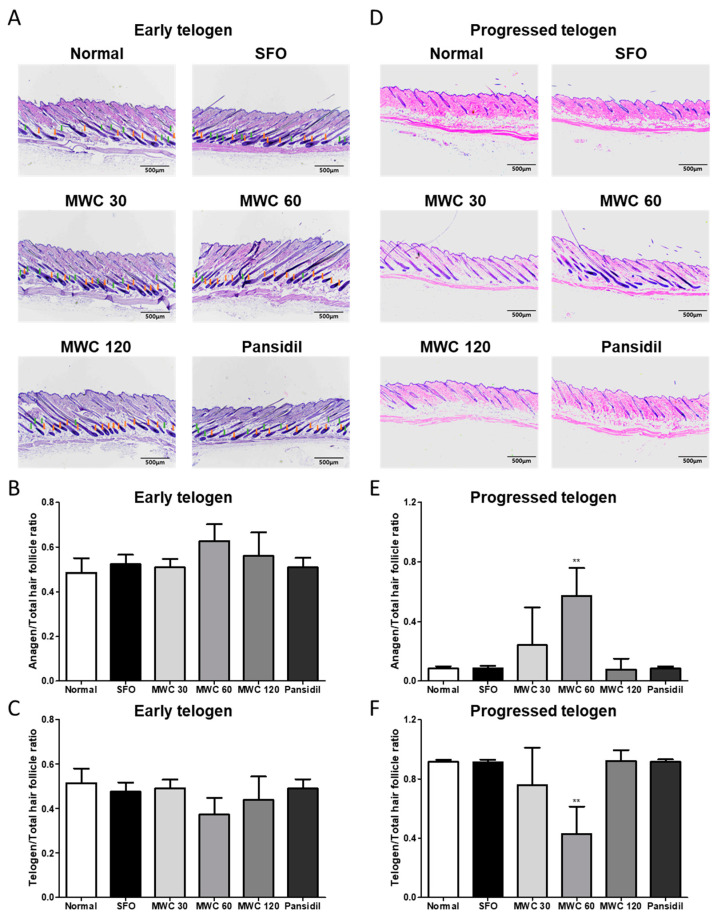
Elongating effect of MWC on hair during the anagen phase in dorsal skin tissues of the anagen-synchronized mouse model. Dorsal skin tissues were stained by the H&E staining method. (**A**) In the early telogen phase, dorsal skin sections were analyzed, and the number of HFs was counted (scale bar = 500 μm). (**B**) The ratios of anagen HFs to total HFs and (**C**) telogen HFs to total HFs were analyzed in the early telogen phase. (**D**) In the late telogen phase, dorsal skin sections were analyzed, and the number of HFs was counted (scale bar = 500 μm). (**E**) The ratios of anagen HFs to total HFs and (**F**) telogen HFs to total HFs were analyzed in the late telogen phase. Results were analyzed using a one-way ANOVA (** *p* < 0.01 vs. control).

Dorsal skin tissues in the early and late telogen phases were obtained to perform H&E tissue staining. The anagen, telogen, and total HFs were analyzed by tissue staining, and the ratios of anagen HFs and telogen HFs to the total HFs were measured. The proportions of anagen and telogen HFs in the early telogen phase were not significantly different between the control and the MWC-intake groups (Figure 6A–C). In the dorsal skin tissues obtained in the late telogen phase, the ratio of the anagen HFs tended to increase, and the ratio of the telogen HFs tended to decrease in the 30 and 60 mg/kg groups in a dose-dependent manner. In particular, a significantly increased anagen HF ratio and decreased telogen HF ratio were observed at 60 mg/kg group compared to the control group (Figure 6D–F).

## 4. Discussion

The hair growth cycle refers to the continuous repetition of the anagen and telogen phases of HFCs, including DPCs [10]. There are two types of factors that affect the hair growth cycles of animals with fur: intrinsic factors (genetic background, sex, etc.) and extrinsic factors (environmental factors, nutrient deficiencies, etc.) [28]. These factors can shorten the anagen phase or prolong the telogen phase, and many studies have been conducted to develop drugs for cycle treatment. The wnt/β-catenin signaling pathway is regulated by various mechanisms [10].

One mechanism that inhibits the telogen phase is the inhibition of DHT, which is generated from testosterone by 5α-reductase [29]. Finasteride and dutasteride are widely used medications that have been reported to act through this mechanism [30]. Spironolactone inhibits the telogen phase by operating as an antagonist of the androgen receptor, the biological target of DHT [31]. However, many people who suffer from hair loss are hesitant to take these medicines because of their adverse and temporary side effects. Another mechanism is the maintenance of the DPCs’ condition through the inhibition of oxidative stress or inflammation [3,4,5,32]. Oxidative stress and inflammation are extrinsic factors that can cause various types of biological damage to HFCs, including aging and the mediated cell death of DPCs, which can eventually shorten the anagen hair cycle and activate the telogen hair cycle [4,5,33]. Severe oxidative stress, through which antioxidants are deregulated, is associated with the aging of DPCs [3]. Furthermore, oxidative stress can accelerate the inflammation of DPCs [4]. The blockage of oxidative stress and inflammation by antioxidant enzymes can prevent the aging of HFCs and eventually enhance the growth factors promoted by the wnt/β-catenin signaling pathway in HFCs [34,35,36,37,38]. High levels of oxidative stress can cause the release of TGFβ2, which is known to inhibit the proliferation of epithelial cells and degrade anagen HFs, eventually contributing to the shortening of the hair cycle [39].

Millet extract and wheat extract can enhance the antioxidative and anti-inflammatory properties of various cells [12,13,14,22,23]. These extracts can also enhance the growth factors in DPCs and HFCs ex vivo [15,16]. We suggest that the MWC can enhance antioxidant enzymes and reduce inflammatory cytokines to protect HFCs. We also considered that the MWC can promote wnt/β-catenin signaling to induce growth factors in HFCs. According to our results, we identified that the MWC treatment increased the mRNA levels of antioxidant factors (catalase and SOD1) and decreased the mRNA levels of inflammatory factors (IL-1β and IL-6) in vivo and in vitro (Figure 2 and Figure 4). We also found that the MWC treatment increased the expression of the hair-growth-related factor genes (wnt10b and β-catenin) and their target growth factor genes (IGF-1, VEGF, and FGF7) in vivo and in vitro (Figure 3 and Figure 5). Interestingly, the TGFβ2 mRNA levels were found to be decreased by the MWC treatment during the late telogen phase in vivo (Figure 5). These alterations suggest that the MWC may activate antioxidant enzymes and suppress the secretion of inflammatory cytokines. Moreover, the MWC may activate growth factors in HFCs, including DPCs, via the wnt/β-catenin signaling pathway. We also identified that the MWC can elongate the anagen phase of dorsal HFs (Figure 6). These actions of the MWC can promote the hair anagen phase and maintain hair growth. Even though we did not confirm the transcriptional activity of β-catenin or the wnt10b signal pathway in protein level or nucleus localization, these phenomena suggest that the MWC can stimulate the hair anagen phase through the induction of growth factors via the wnt/β-catenin signaling pathway. Various medications affecting the hair growth cycle can induce an imbalance of sexual hormones and their related genes, such as DHT, testosterone, and 5α-reductase. Furthermore, the effects of these medications weaken due to drug resistance when the administration is continued for long periods. Moreover, these effects are lost when the administration is discontinued. To determine whether the MWC may cause hormonal issues, we analyzed the contents of dihydrotestosterone in the blood after the administration of the MWC for the entire period of this study. However, we confirmed that the MWC treatment did not suppress the DHT levels in mice (Figure S2).

## 5. Conclusions

This study was performed to identify the effect of the MWC on hair health in animal models. These results suggest that the MWC may improve hair health in various ways, such as through antioxidative and anti-inflammatory actions and the promotion of hair growth factors via the wnt/β-catenin signaling pathway. Thus, the MWC has potential as a hair health treatment without any adverse side effects, such as hormone imbalance.

## Figures and Tables

**Table 1:** The sequence primer of target genes.

Primer	Sequence	
GAPDH	Forward	5′-TGACCTCAACTACATGGTCTACA-3′
	Reverse	5′-CTTCCCATTCTCGGCCTTG-3′
Catalase	Forward	5′-AGCGACCAGATGAAGCAGTG-3′
	Reverse	5′-TCCGCTCTCTGTCAAAGTGTG-3′
SOD1	Forward	5′-AACCAGTTGTGTTGTCAGGAC-3′
	Reverse	5′-CCACCATGTTTCTTAGAGTGAGG-3′
IL-1β	Forward	5′-TTCAGGCAGGCAGTATCACTC-3′
	Reverse	5′-GAAGGTCCACGGAAAGACAC-3′
IL-6	Forward	5′-TGAACAACGATGATGCACTTG-3′
	Reverse	5′-CTGAAGGACTCTGGCTTTGTC-3′
TNF-α	Forward	5′-CAGGCGGTGCCTATGTCTC-3′
	Reverse	5′-CGATCACCCCGAAGTTCAGTAG-3′
IGF-1	Forward	5′-AGGAAGTACATTTGAAGAACGCAAGT-3′
	Reverse	5′-CCTGCGGTGGCATGTCA-3′
VEGF	Forward	5′-GTGGACATCTTCCAGGAGTACC-3′
	Reverse	5′-TGTTGTGCTGTAGGAAGCTCAT-3′
FGF-7	Forward	5′-CTGTCGAACACAGTGGTACCTG-3′
	Reverse	5′-CCAACTGCCACTGTCCTGATTTC-3′
SOX-2	Forward	5′-GAGCTTTGCAGGAAGTTTGC-3′
	Reverse	5′-GCAAGAAGCCTCTCCTTGAA-3′
β-catenin	Forward	5′-AAAGCGGCTGTTAGTCACTGG-3′
	Reverse	5′-CGAGTCATTGCATACTGTCCAT-3′
wnt10b	Forward	5′-CTCGGGATTTCTTGGATTCCAGG-3′
	Reverse	5′-GCCATGACACTTGCATTTCCGC-3′
TGFβ2	Forward	5′-AAGAAGCGTGCTTTGGATGCGG-3′
	Reverse	5′-ATGCTCCAGCACAGAAGTTGGC-3′

## Data Availability

Data will be made available on request.

## Supplementary Materials

The following supporting information can be downloaded at https://www.mdpi.com/article/10.3390/nu15204411/s1, Figure S1: Design for the experiment of MWC on hair health through the anagen-synchronized mice model. Figure S2: The lack of suppression of DHT levels in the plasma of whole blood by MWC in the anagen-synchronized mouse model.
