# Supplementary material for: Effects of the Complex of Panicum miliaceum Extract and Triticum aestivum Extract on Hair Condition
Source: Nutrients. 2023 Oct 18;15(20):4411. doi: 10.3390/nu15204411 (PMC10609892; doi:10.3390/nu15204411)
Supplement: Supplementary file 1 [file nutrients-15-04411-s001.zip › nutrients-2634222-supplementary.pdf]

Supplement materials and methods

DHT detection

DHT levels were measured in the plasma of mice in the late telogen phase. Plasma was prepared to detect DHT by immediately collecting the whole blood of mice after necropsy. DHT detection was performed using a DHT ELISA kit (Abcam, IA, USA) following the manufacturer’s protocol.

Supplement Figures

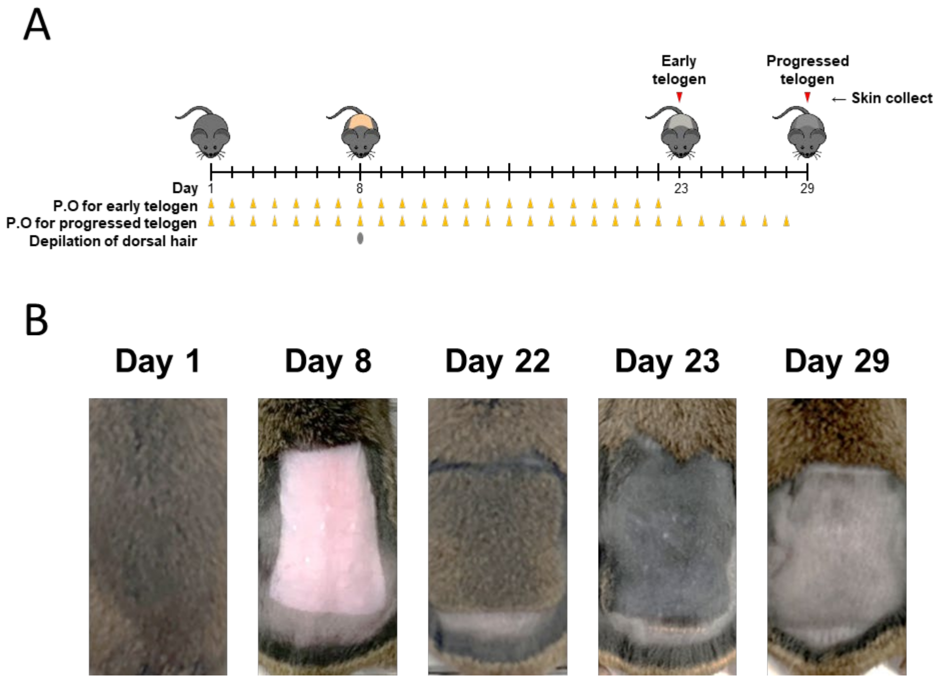

Supplement Figure S1 Design for the experiment of MWC on hair health through the anagen-synchronized mice model. (**A**) C3H mice, 6.5 weeks old, were fed 30–120 mg/kg of MWC, 120 mg/kg of SFO, and 400 mg/kg of Pansidil every day for 22 or 28 days, and tissues were collected from early or late telogen phase dorsal skin, respectively. The test articles were orally administered every day until the collection of the dorsal skin. (**B**) The dorsal hair was depleted on Day 8, and then the hair was shaved on Day 23 or 29 to measure the amount of anagen and telogen HFs.

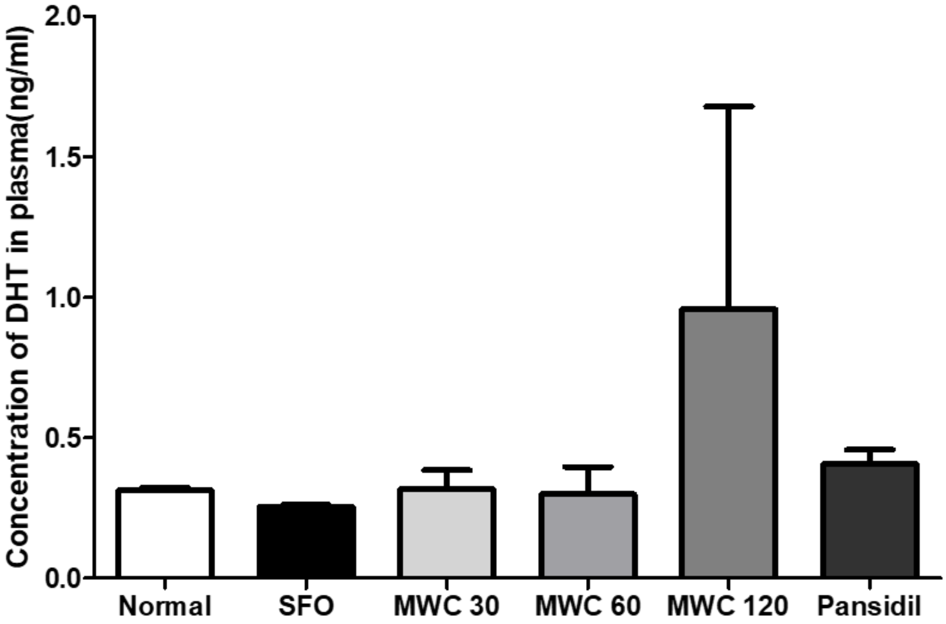

Supplement Figure S2. The lack of suppression of DHT levels in the plasma of whole blood by MWC in the anagen-synchronized mouse model. DHT levels were measured from the plasma of whole blood in C3H/HeN mice that were administered MWC until Day 29. Results were analyzed using a student's t-test.
